# Evidence synthesis to policy: development and implementation of an impact-oriented approach from the Eastern Mediterranean Region

**DOI:** 10.1186/s12961-023-00989-5

**Published:** 2023-06-01

**Authors:** Fadi El-Jardali, Racha Fadlallah, Lama Bou Karroum, Elie A. Akl

**Affiliations:** 1 Department of Health Management and Policy, Faculty of Health Sciences, American University of Beirut, Beirut, Lebanon; 2 Center for Systematic Reviews On Health Policy and Systems Research (SPARK), American University of Beirut, Beirut, Lebanon; 3 Knowledge to Policy (K2P) Center, American University of Beirut, Beirut, Lebanon; 4 Department of Health Research Methods, Evidence, and Impact (HEI), McMaster University, Hamilton, ON Canada; 5 Department of Internal Medicine, American University of Beirut Medical Center, Beirut, Lebanon

**Keywords:** Evidence synthesis, Approach, Policy-relevant, Systematic review, Impact, Eastern Mediterranean Region, Impact, Evidence-to-policy, Knowledge translation, Lebanon

## Abstract

**Background:**

Despite the importance of evidence syntheses in informing policymaking, their production and use remain limited in the Eastern Mediterranean region (EMR). There is a lack of empirical research on approaches to promote and use policy-relevant evidence syntheses to inform policymaking processes in the EMR.

**Objective:**

This study sought to describe the development of an impact-oriented approach to link evidence synthesis to policy, and its implementation through selected case studies in Lebanon, a middle-income country in the EMR.

**Methods:**

This study followed a multifaceted and iterative process that included (i) a review of the literature, (ii) input from international experts in evidence synthesis and evidence-informed health policymaking, and (iii) application in a real-world setting (implementation). We describe four selected case studies of implementation. Surveys were used to assess policy briefs, deliberative dialogues, and post-dialogue activities. Additionally, Kingdon’s stream theory was adopted to further explain how and why the selected policy issues rose to the decision agenda.

**Results:**

The approach incorporates three interrelated phases: (1) priority setting, (2) evidence synthesis, and (3) uptake. Policy-relevant priorities are generated through formal priority setting exercises, direct requests by policymakers and stakeholders, or a focusing event. Identified priorities are translated into focused questions that can be addressed via evidence synthesis (phase 1). Next, a scoping of the literature is conducted to identify existing evidence syntheses addressing the question of interest. Unless the team identifies relevant, up-to-date and high-quality evidence syntheses, it proceeds to conducting SRs addressing the priority questions of interest (phase 2). Next, the team prepares knowledge translation products (e.g., policy briefs) for undertaking knowledge uptake activities, followed by monitoring and evaluation (phase 3). There are two prerequisites to the application of the approach: enhancing contextual awareness and capacity strengthening. The four case studies illustrate how evidence produced from the suites of activities was used to inform health policies and practices.

**Conclusions:**

To our knowledge, this is the first study to describe both the development and implementation of an approach to link evidence synthesis to policy in the EMR. We believe the approach will be useful for researchers, knowledge translation platforms, governments, and funders seeking to promote evidence-informed policymaking and practice.

## Background

There is growing recognition that the use of evidence in health policymaking can strengthen health systems, accelerate progress on achieving the Sustainable Development Goals (SDGs), and improve population health [1–5]. Most recently, the COVID-19 pandemic has increased demands for evidence syntheses to inform critical policy decisions and enhance accountability and public trust in decision-makers [2, 6].

Evidence syntheses use formal explicit rigorous methods to provide an account of the totality of what is known from preexisting research [7]. They can be useful to identify gaps in knowledge, establish an evidence base for best-practice guidance, or help inform policymakers and practitioners [8]. A systematic review (SR) is the most common type of evidence synthesis. It is defined as a “review of research literature using systematic, explicit, and accountable methods” [9]. Other types include rapid reviews, gap maps, overviews of systematic reviews, and scoping reviews [10]. Systematic reviews and other types of evidence syntheses are increasingly recognized as having a key role in informing the decision-making process [11, 12]. Policy-relevant evidence syntheses can help clarify policy problems or provide evidence about the effectiveness or implementation of health policies and health systems interventions, while considering the diversity of people and contexts [13]. There are many types of outputs that use evidence synthesis, such as policy briefs and clinical practice guidelines [14].

Despite their importance in informing policymaking, the production and use of evidence syntheses remain limited in the Eastern Mediterranean region (EMR) [15–17]. A study assessing the profile of SR production in 41 low- and middle-income countries (LMICs) found that the EMR is among the lowest in terms of SR production, with only 10% of the total included studies produced by a corresponding author based in the EMR [17]. These findings align with a situational analysis of SRs in the EMR, which revealed a very low production of SRs in the region [15]; study findings also showed a gap in the production of policy-relevant SRs, in which only 19.1% of the identified SRs matched priorities identified by policymakers and stakeholders in the EMR. A more recent overview of systematic reviews published between 2008 and 2016 on population health in EMR countries found that, while publication of SRs has increased over time, the topics addressed in the SRs do not appear to match the disease burden in the region [18]. The limited production of policy-relevant evidence syntheses has been attributed to the poor interaction between researchers and policymakers. For example, in a survey of policymakers from the EMR, around 65% of respondents indicated that SRs on high priority issues were rarely disseminated to policymakers [19]. Additionally, policymakers highlighted the need for better packaging of research results to assist in evidence-informed policymaking [20]. Similarly, a survey of over 200 researchers and health research institutions, respectively, from the EMR revealed that very few produced policy briefs tailored to policymakers, interacted with policymakers and stakeholders in priority settings, or involved them in their research [21, 22].

There has been mounting interest over the years in the use of frameworks, approaches, and models to gain insights into mechanisms by which uptake and use of evidence is more likely to succeed [23–28]. Nonetheless, the use of evidence in policymaking has not reached the same state of maturity and development as in the clinical field. Many of the existing approaches and frameworks focus on primary research or research in general; lack direct links between research and its use; do not put emphasis on context that is critical for informing policymaking; or fail to address capacity needs, stakeholder involvement, and monitoring and evaluation as key aspects of the approach [24, 25, 28, 29]. Furthermore, empirical evidence to support testing of the frameworks is limited [29]; where such evidence exists, it is usually from high-income countries, with lack of evidence base on their applicability to LMICs [30].

In the EMR, there is a lack of empirical research on approaches to promote and use policy-relevant evidence syntheses to inform policymaking processes. In an effort to address the aforementioned gaps, we developed an “impact-oriented approach to link evidence synthesis to policy,” hereby referred to as “approach.” In this paper, we describe the approach followed by its implementation through selected case studies in Lebanon, a middle-income country in the EMR. It is worth noting that at the time of its development and implementation, Lebanon was classified by the World Bank as an upper middle-income country but since then has suffered a series of crises and is now classified as a low middle-income country [31].

## Methods

**Fig. 1:**
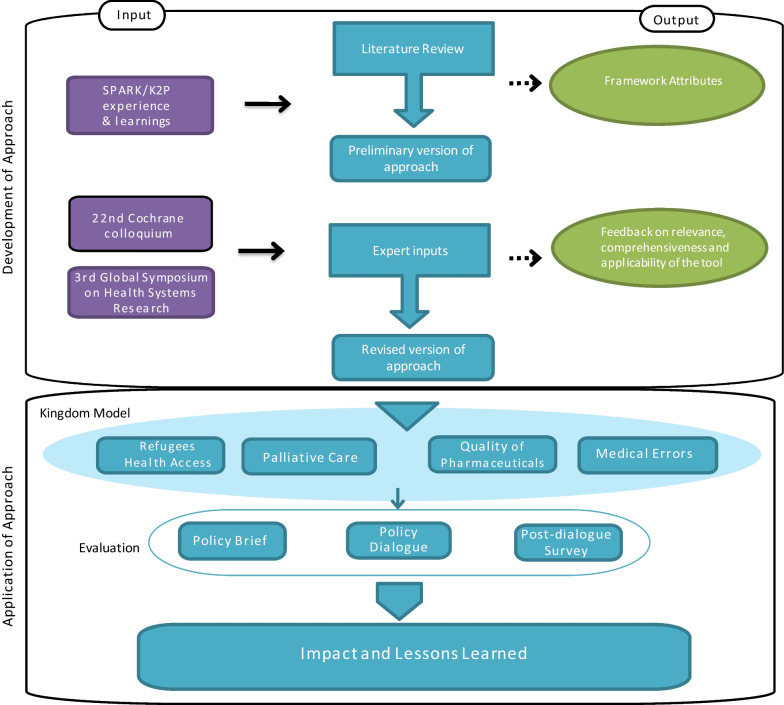
Summary of steps

We followed a multifaceted and iterative process that included (i) a review of the literature, (ii) input from international experts in evidence synthesis and evidence-informed health policymaking, and (iii) application in a real-world setting (implementation) through selected case studies (Fig. 1).

(i) Review of literature

We conducted a literature review to identify approaches/frameworks/models/strategies associated with evidence synthesis knowledge translation and evidence-informed health policymaking. We used the following combination of terms to search Medline and PubMed: (research OR evidence OR knowledge) AND (synthesis OR translation OR uptake OR implementation OR use) AND (approach OR framework OR model OR theory). The articles were screened by two members of the team for relevance. We included papers that focused on promoting evidence-informed health policymaking, with priority given to evidence synthesis. We generated a preliminary list of framework attributes from all relevant papers. The attributes were reviewed and discussed with all members of the team, taking into consideration the experience and learning from two collaborating knowledge centers established at the American University of Beirut (AUB) in Lebanon to address high priority policy issues. First, the Center for Systematic Reviews of Health Policy and Systems Research (SPARK) specializes in the production of policy-relevant systematic reviews and other evidence synthesis products in the field of health policy and systems research. Second, the Knowledge to Policy (K2P) center specializes in knowledge translation products (leveraging existing evidence synthesis), knowledge uptake activities, and impact assessment. Both SPARK and K2P form a unique collaboration to promote evidence-informed health policymaking in Lebanon and the EMR. A preliminary version of the approach was subsequently generated.

(ii) Input from international experts

We sought input from two groups of experts. The first group included participants in a workshop on an approach and tool for prioritizing questions for evidence synthesis in health policy and systems research at the 22nd Cochrane Colloquium in Hyderabad, India.^1^ The same version of the approach was also presented to a second group of participants at the Third Global Symposium on Health Systems Research held in Cape Town, South Africa.^2^ The first group included systematic reviewers and evidence synthesis specialists, while the second group invited health policy and systems researchers as well as policymakers and funders in the field of health policy and systems research. Each expert group session involved 18–20 participants and lasted 1.5 h. Participants were split into small working groups, with assigned facilitators to elicit participants’ input on the clarity, comprehensiveness, and applicability of the framework to their context. Thorough notes were taken of the discussions that took place. The research team used the qualitative feedback from both groups to revise the approach.

(iii) Application in a real-world setting

The approach was implemented by SPARK and K2P centers to address high-priority policy issues. For the purpose of this study, we focused on the following four issues: (1) enhancing access of Syrian refugees to basic healthcare services; (2) promoting quality of pharmaceuticals; (3) addressing medical errors in hospitals; and (4) integrating palliative care into the healthcare system. These have been selected for two main reasons: the first being pragmatic considerations, including the availability of relevant documents on the cases, and the second being that these issues represented a diversity in the source of prioritization, conduct of evidence synthesis, and impact achieved. The uniqueness of each case study as well as intersection points will enable cross-comparisons across the different components of the framework, which in turn will help build a more comprehensive understanding of the activities and key learnings for enhancing these activities under each component of the approach.

To evaluate “impact” of research on policy, we undertook both formative and summative evaluations. For the formative part, the following activities and outputs were evaluated using standardized tools: policy briefs, deliberative dialogues, and post-dialogue follow-up. Responses were analyzed descriptively.

- Policy brief: we adapted the evaluation survey from Lavis et al. and circulated it to participants at the dialogue and followed up with reminder emails a few days later [32]. The survey consisted of ten items rating how helpful different aspects of the brief were, as well as how well the brief achieved its purpose of presenting the available research evidence to inform the policy dialogue. The survey used a scale from 1 to 7 (1 being very unhelpful, 4 being neutral, and 7 being very helpful).
- Deliberative dialogue: we also adapted the survey from Lavis et al. and circulated it to participants at the end of the dialogue [32]. The survey consisted of eight items rating how helpful they found different aspects of the dialogue, as well as how well the dialogue achieved its purpose of supporting a full discussion of relevant considerations about the problems and its policy approach to inform action. The survey uses the same scale as the policy brief evaluation survey.
- Post-dialogue survey: 6 months following the dialogue, we circulated a short questionnaire to 4–6 key participants purposively selected from different agencies that championed the issue of interest, each in their own capacity. The questionnaire aimed to follow-up on the deliberations that took place, track progress, and identify implementation issues pertaining to achieving impact.

For the summative evaluation, we applied the “theory of change” to examine whether the set of activities and outputs presented within the different phases of the approach collectively led to impact on select policymaking processes. Theory of change allows for multiple pathways, levels of interventions, and feedback loops that better reflect the reality of how complex interventions achieve their impact [33]. It can therefore address attribution, i.e., the role played by the approach more generally in informing the policymaking process.

To further explain how and why policy issues rise to the decision agenda, we adopted Kingdon’s multiple stream model [34]. The model consists of three independent streams: problems, policies, and politics. It argues that a “policy window” opens only when the three streams converge, propelling governments to act. The problems stream contains the broad problems facing societies, some of which become identified as issues that require public attention. The policy stream refers to the set of policy alternatives that researchers and other stakeholders propose to address national problems. The politics stream consists of political transitions, national mood, elections, or pressure from interest groups [35].

## Results

This section begins with a description of the approach followed by its implementation to selected priority policy issues.

### Description of the approach

**Fig. 2:**
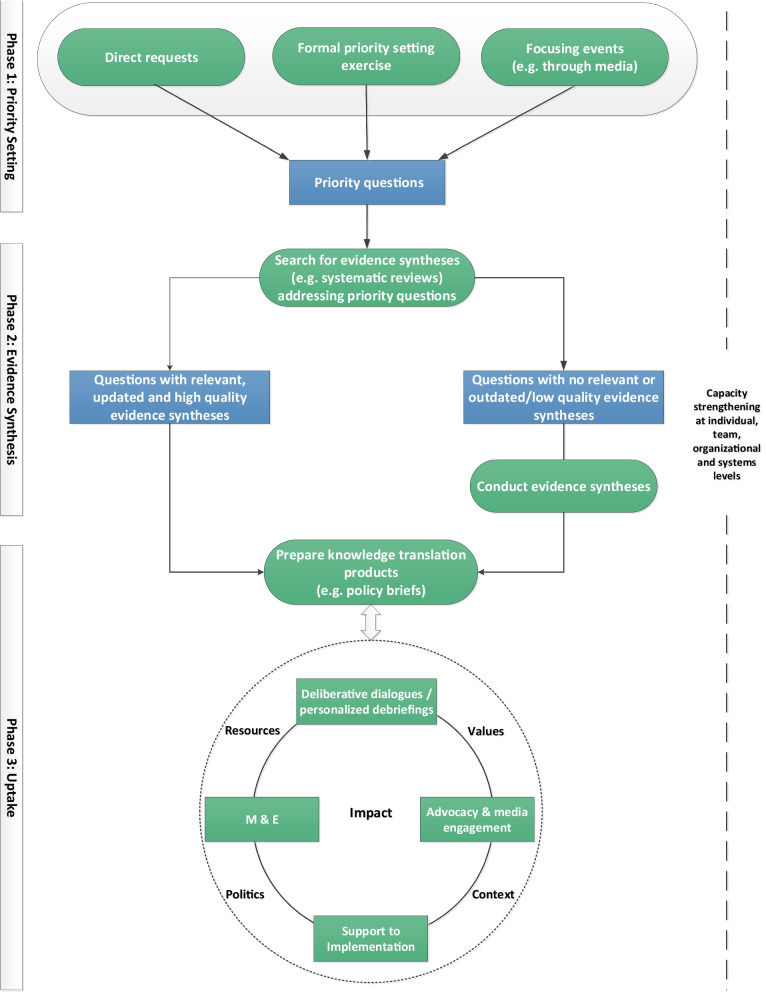
Impact-oriented approach to link evidence synthesis to policy

The approach incorporates three interrelated phases: (1) priority setting, (2) evidence synthesis, and (3) uptake. The prerequisites to the implementation of the approach include enhancing contextual awareness and capacity strengthening encompassing all three phases (Fig. 2). The approach encourages researchers, policymakers, and stakeholders to closely interact at each phase to achieve the desired impact.

A description of each of phase of the approach is further provided below:

- Phase 1, priority setting: a key starting point to inform policymaking process is to identify policy-relevant priorities. Policy-relevant priorities for which evidence syntheses are needed can be generated through formal priority setting exercises, direct requests from policymakers and stakeholders, or a focusing event, for example, through media coverage. The priority setting exercises can benefit from published tools [36, 37]. Identified priorities are translated into clearly focused priority questions that can be addressed via evidence synthesis.
- Phase 2, evidence synthesis: a scoping of the literature is conducted to identify existing evidence syntheses (e.g., systematic reviews) addressing the prioritized question of interest. The identification of relevant, up-to-date, and high-quality evidence syntheses would help avoid duplication of efforts. When relevant, up-to-date, and high-quality evidence syntheses (as assessed using AMSTAR or ROBIS tools) are identified, the K2P Center team proceeds directly to preparing knowledge translation products (phase 3). Otherwise, the SPARK Center team proceeds with conducting evidence syntheses addressing the question of interest [38].
- Phase 3, uptake: this phase involves preparing knowledge translation products, undertaking knowledge uptake activities, and monitoring and evaluation to achieve impact. Central to this phase are contextual factors that influence the degree of uptake and use of evidence in policymaking.
  Knowledge translation products are prepared, drawing on evidence syntheses, particularly systematic reviews. One widely used product is the policy brief, which brings together global research evidence (from SRs), local evidence, and context-specific knowledge to inform deliberations about health policies and programs. An important feature of the policy brief is the involvement of end users in its development and revision to help capture tacit knowledge and contextualized evidence. Additional knowledge translation products include briefing notes and rapid response documents. The knowledge translation products are disseminated to target policymakers and stakeholders using a range of uptake activities. A widely used uptake activity is the deliberative stakeholder dialogue, which brings together research evidence from the knowledge translation product alongside local context, experiences, views, and tacit knowledge of stakeholders who will be involved in or affected by decisions about the issue to increase the prospects of using the evidence in policy [39]. A dialogue summary, which provides a summary of the key deliberations and a roadmap for action (taking into consideration resources, politics, context), is subsequently prepared and disseminated to all participants. Additional uptake activities include personalized debriefings for selected stakeholders, and close follow-up with policy implementers (i.e., support to implementation) to help overcome potential barriers to implementation. Media engagement, citizen consultations, and advocacy activities can also be leveraged to mobilize public opinion and pressure for policy change. Monitoring and Evaluation (M&E) is iterative and occurs at both the process and outcome levels to determine whether and how evidence from the suites of activities was used (or not) to inform policymaking and practice. At the process level (i.e., formative evaluation), the research team evaluates the priority-setting process (for formal priority-setting exercises), the knowledge translation products (e.g., policy briefs) and uptake activities (e.g., deliberative dialogues) using standardized evaluation surveys to identify areas of strength and those requiring improvement. At the outcome level (i.e., summative evaluation), post-dialogue surveys or interviews are conducted with key stakeholders from different agencies that championed the issue at least 6 months after the dialogue. The purpose is to follow up on the deliberations that took place as well as identify actions taken by stakeholders and implementation challenges they may have encountered in translating the policy options or elements that were discussed at the dialogue into policy and action. Self-reported evidence is further validated through publications, reports, archival records, and media articles.

#### Prerequisites

There are two prerequisites to the application of the approach.

First, it is important to start with a preparatory phase to understand the political context and the receptiveness of policymakers and stakeholders to evidence-informed health policymaking. The preparatory phase is also critical to enhance contextual and policy awareness [40]. For example, prior to implementation of the approach by SPARK and K2P centers, we surveyed policymakers, stakeholders, and researchers to elicit their input on the role of evidence in policymaking, as well as to identify barriers to and facilitators of the use of evidence in policymaking.

Second, increasing the use of evidence in policymaking requires strengthening capacity on both the supply side, in terms of capacity for generation and uptake of policy-relevant evidence, and the demand side, in terms of the demand for and use of evidence by policymakers for policy decisions. Efforts should aim to target different levels, including both individual and organizational levels, to ensure sustainability of evidence-informed policymaking and practice. For this purpose, we have adapted the capacity-building framework by Oliver et al. to develop capacity at the individual, team, organizational, and systems levels [38].

### Implementation of the approach

We present here four case studies in which we implemented the approach. Table 1 and the subsequent sections describe the process adopted and the impact achieved for each case study. The case studies illustrate how evidence produced from the suites of activities depicted in the approach was used to inform health policies and practices for selected priority topics.

**Table 1:** Overview of the process

	Syrian refugees	Quality of pharmaceuticals	Medical errors	Palliative care
Priority setting				
Formal priority setting exercise	✓	✓		
Direct request from stakeholders		✓		✓
Focusing event			✓	
Evidence synthesis				
Conducting new systematic reviews (or other evidence syntheses)	✓	✓		
Leveraging existing evidence syntheses			✓	✓
Knowledge translation				
Knowledge translation products	✓	✓	✓	✓
Deliberative dialogues	✓	✓	✓	✓
Post-dialogue summaries	✓	✓	✓	✓
Personalized debriefings for selected stakeholders		✓	✓	
Media engagement (newspaper articles, media appearances)		✓		✓
Advocacy/creation of coalition network				✓
Follow-up on implementation	✓		✓	
Materials created for policy implementers	✓		✓	

Two of the topics, Syrian refugees and quality of pharmaceuticals, were identified through formal priority setting meetings, while palliative care was generated through direct request from stakeholders, and the topic on medical errors was shaped by a focusing event in the country (phase 1). A quick scoping of the literature did not identify any updated and high-quality SRs for the two priorities identified through the formal priority setting meetings; thus, the SPARK team proceeded with conducting SRs addressing the topics (phase 2), and these were subsequently incorporated into knowledge translation products, specifically policy brief and briefing note. As for the other two topics for which there were preexisting updated and high-quality SRs, the K2P Center team directly proceeded with preparing knowledge translation products (phase 3).

A range of uptake activities was undertaken to promote the use of the evidence in health policymaking and practice (phase 3). While some were common across all four case studies, such as convening deliberative dialogues (pre-informed by the knowledge translation products), there were also some notable variations. For instance, follow-up on implementation, including generation of materials to support the implementation process, was conducted for both Syrian refugees (through the development of Terms of Reference (TORs) for the refugee health coordinator to be recruited by the Lebanese Ministry of Public Health) and medical errors (through the establishment of national standards for patient safety including training requirements for the hospital accreditation program). For palliative care, stakeholders created a coalition of non-governmental organizations (NGOs) and engaged in additional advocacy efforts to achieve the desired impact. The issue of quality of pharmaceuticals involved personalized briefings with selected stakeholders (representatives from Ministry of Public Health and Deans of Schools of Medicine) and media engagement (e.g., development of media bytes) to prompt stakeholders to act on key findings from the deliberations.

In all four case studies, researchers, policymakers, and stakeholders interacted at each phase of the approach, from identifying priorities to shaping priority questions (and co-production of the reviews with a policymaker from the Ministry of Public Health for the case study on quality of pharmaceuticals) to framing the scope of knowledge translation products, to engaging in deliberative dialogues, and finally to monitoring and evaluation to achieve the desired impact.

Different types of evidence were collectively used throughout the approach. While scientific knowledge were derived from research (for the evidence syntheses) and local data (for the knowledge translation product), tacit knowledge was derived from stakeholder interviews as well as elicited through deliberative dialogues and personalized debriefings with policymakers and stakeholders.

A total of 26 and 29 participants, respectively, completed the policy brief and deliberative dialogue evaluation surveys for two of the four case studies (medical errors and quality of pharmaceuticals). Results of the evaluation of the policy brief and deliberative dialogue are presented in Table 2. Average scores were positive and exceeded 5 (out of 7) for all features of the policy brief. The specific aspects that were found most helpful by participants were the graded-entry format that the brief employed (a list of key messages, executive summary, and a full report), the fact that the brief described different features of the problem and three elements of an approach for addressing it, and that the brief described the context for the issue being addressed. Compared with the other key features of the briefs, “not concluding with recommendations” and “taking local applicability considerations into account when discussing the research evidence” were judged less favorably by respondents. The results of the evaluation of the deliberative dialogues also yielded favorable results, with average scores exceeding 5 (out of 7) on all features. Aspects deemed helpful were the fact that the dialogue was informed by a pre-circulated Policy Brief/Briefing Note, it engaged a facilitator to assist with the deliberations, and it allowed for frank and off-the-record deliberations. However, “not aiming for consensus” was viewed less favorably than any other key feature.

**Table 2:** Survey evaluation of policy briefs and deliberative dialogues

Policy briefs	Medical errors	Quality of pharmaceuticals	Total score*
1. The policy brief described the context for the issue being addressed	6.55	6.5	6.53
2. The policy brief described different features of the problem, including (where possible) how it affects particular groups	6.63	6.5	6.57
3. The policy brief described elements of an approach for addressing the problem	6.53	6.5	6.52
4. The policy brief described what is known on the basis of synthesized research evidence about each of the elements and where there are gaps in what is known	6.6	6.4	6.50
5. The policy brief described key implementation considerations	6.1	6	6.05
6. The policy brief took quality considerations into account when discussing the research evidence	6.35	6.4	6.38
7. The policy brief took local applicability considerations into account when discussing the research evidence	5.78	5.8	5.79
8. The policy brief did not conclude with particular recommendations	5.57	5.7	5.64
9. The policy brief employed a graded-entry format (e.g., a list of key messages and a full report)	6.65	6.7	6.68
10. The purpose of the policy brief was to present the available research evidence on a high-priority policy issue to inform a policy dialogue in which research evidence would be just one input to the discussion. How well did the policy brief achieve its purpose?	6.74	6.3	6.52

Deliberative dialogues	Medical errors	Quality of pharmaceuticals	Total score*
1. The stakeholder dialogue was informed by a pre-circulated Policy Brief/Briefing Note	6.76	6.7	6.73
2. The stakeholder dialogue was informed by discussion about the full range of factors that can inform how to approach a problem, possible elements of an approach for addressing it, and key recommendations	6.47	6.7	6.59
3. The stakeholder dialogue brought together many parties who could be involved in or affected by future decisions related to the issue	6.53	6.2	6.36
4. The stakeholder dialogue aimed for fair representation among policymakers, stakeholders, and researchers	6.59	6.2	6.39
5. The stakeholder dialogue engaged a facilitator to assist with the deliberations	6.71	6.6	6.65
6. The stakeholder dialogue allowed for frank, off-the-record deliberations by following the Chatham House rule: “Participants are free to use the information received during the meeting, but neither the identity nor the affiliation of the speaker(s), nor that of any other participant, may be revealed.	6.63	6.6	6.61
7. The stakeholder dialogue did not aim for consensus	6.36	5.6	5.98
8. The purpose of the stakeholder dialogue was to support a full discussion of relevant considerations (including research evidence) about a high-priority policy issue to inform action. How well did the stakeholder dialogue achieve its purpose?	6.69	6.2	6.44

^*^Maximum score is 7 for each variable

Post-dialogue follow-up with key selected stakeholders revealed positive attitudes toward research evidence of the type discussed at the dialogues, as well as strong intentions to use research evidence and actual adoption and implementation of some of the evidence-based policy options. For the latter, documentation review and media analysis were also conducted to further capture and document impact.

Boxes 1, 2, 3, 4 describe the process adopted and the impact achieved for each of the four selected case studies.

Box 1: Process adopted and impact achieved for the topic of promoting Access to essential health care services for Syrian refugees **Issue**: Syrian refugees in Lebanon face a high burden of communicable and non-communicable diseases. Providing health services to this large number of refugees is a real challenge, given limited capacity of the health system and the preexisting economic crisis in Lebanon.

**Priority setting**: Formal priority-setting exercise brought together 54 policymakers who were representatives from the Ministry of Public Health (MOPH), Lebanese National Council for Research, World Health Organization (WHO) Country Office, United Nations Development Programme, United Nations High Commissioner for Refugees (UNHCR), United Nations Relief and Works Agency for Palestine Refugees in the Near East, International Organization for Migration, and UK Department for International Development, in addition to academics and researchers. Participants shaped and prioritized the question of limited coordination between organizations and agencies providing health services to refugees.

**Evidence synthesis**: Production of two systematic reviews addressing the questions identified in the priority setting step:

- Addressing Mechanisms and Models of Coordination between Organizations, Agencies and Bodies Providing or Financing Health Services in Humanitarian Crises: A Systematic Review
- Coordinating the Provision of Health Services in Humanitarian Crises: A Systematic Review of Suggested Models

**Knowledge translation product:** A briefing note titled “Promoting Access to Essential Health Care Services for Syrian Refugees in Lebanon.” Findings from the two systematic reviews were incorporated into the briefing note. In addition to key findings from the systematic reviews, the briefing note synthesized other relevant global and local research evidence, and contextualized the evidence to the Lebanese health system. Policymakers and stakeholders were involved in shaping and framing the outline for the briefing note.

**Knowledge uptake activities:** A national policy dialogue, titled “Promoting access to essential health care services for Syrian refugees in Lebanon” informed by the briefing note. The dialogue was attended by 28 policymakers and stakeholders involved in providing and/or financing health services for refugees in Lebanon as well as representatives of the refugee population. A post-dialogue summary was prepared and disseminated to all participants. Follow-up on implementation was conducted with key selected policymakers and stakeholders.

**Impact:**

Recruitment of a Refugee Health Response Coordinator at the Ministry of Public Health. The coordinator has helped the Ministry of Public Health to establish partnerships with local and international agencies, donors, and academic institutions; develop a refugee health information system; and assist in overseeing the development and implementation of the comprehensive strategic plan for responding to the health needs of Syrian refugees in Lebanon (informed by the briefing note). The coordinator reconvened all of the stakeholders who participated in the policy dialogue to form a Health Steering Committee. The coordinator developed the Health Response Strategy, with guidance from a number of officials and policymakers in the MOPH; this document was released in late 2015 and then updated in 2016. By developing this national strategy, the MOPH assumed a leadership role in coordinating and guiding health response efforts.

**Reference Document**: “This work has made significant progress in addressing the recommendations above and in promoting better access to basic health-care services to Syrian refugees,” says Dr Ammar (Director General at Ministry of Public Health, Lebanon, at the time). https://www.who.int/evidence/resources/publication/en/

Health response strategy: a new approach in 2016 & beyond. Beirut: Lebanese Ministry of Public Health; 2015 (http://www.moph.gov.lb/userfiles/files/Strategic%20Plans/HRS-DRAFT8.pdf),

Box 2: Process adopted and impact achieved for the topic of promoting quality of pharmaceuticals in Lebanon **Issue**: Per capita expenditure on pharmaceuticals in Lebanon is the highest in the Eastern Mediterranean Region and one of the highest globally. This is further exacerbated by inappropriate drug prescribing patterns (due to poor regulation of physician–industry interactions); dominance of brand name drugs (> 70%); and reported cases of counterfeit and substandard drugs.

**Priority setting**: Two-step process:

Selection of topics: Considered the priorities from two previously conducted priority-setting exercises followed by informal consultations with selected policymakers and stakeholders. One of the topics that emerged was on strengthening pharmaceutical policies in Lebanon.

Selection of questions: Formal priority-setting exercise bringing together 15 policymakers and stakeholders including representatives from the Ministry of Public Health; representatives from different orders and syndicates; representatives from academic medical centers, medical schools, and schools of pharmacies; healthcare managers and academic researchers; representatives from international and national agencies; and representatives from insurance companies. Participants shaped and prioritized the following two questions:

- Strategies to reduce counterfeit and substandard drugs
- Strategies to improve prescribing of drugs.

**Evidence synthesis**: Production of a series of systematic reviews addressing the questions identified in the priority-setting step. One of the policymakers from the priority-setting phase was involved in co-production of the first systematic review (interventions to combat or prevent drug counterfeiting):

- Effectiveness of interventions to combat or prevent drug counterfeiting
- Legislative, educational, policy & other interventions targeting physician-pharmaceutical industry interactions
- Association between physician-pharmaceutical industry interactions and clinical practices
- Extent and type of interactions of physicians with pharmaceutical industry.

**Knowledge translation product:** Policy brief titled “Improving the Quality and Prescribing of Pharmaceuticals in Lebanon.” Findings from the systematic reviews were incorporated into the policy brief. In addition to these key findings, the policy brief gathered and synthesized other relevant global and local research evidence, and contextualized the evidence to the Lebanese health context. Key policymakers and stakeholders were involved in shaping and framing the outline for the policy brief.

**Knowledge uptake activities:** A national policy dialogue titled “Improving the Prescribing Pattern and Quality of Pharmaceutical Drugs in Lebanon,” which was attended by 25 policymakers, practitioners, different orders, and syndicates and researchers involved or affected by pharmaceutical sector in Lebanon. A post-dialogue summary was disseminated to all participants. Personalized briefings were conducted with selected policymakers from the MoPH and Deans of Schools of Medicine. Media engagement was leveraged to maintain momentum and pressure for change.

**Impact:**

- Revision of Code of Ethics for Pharmaceutical Promotion
- Integration of clinical pharmacy services in health care organizations (as part of hospital accreditation standards)
- Revision of undergraduate medical curricula at AUB to incorporate education about industry marketing techniques

**Reference Document**: The code is ready to be launched after finalization as a result of a wide participatory process that lasted for 2 years with all relevant stakeholders https://www.moph.gov.lb/en/view/4753/drugs-ethical-standards-

The revised hospital accreditation standard includes a new chapter on clinical pharmacy services: https://www.moph.gov.lb/en/Pages/3/20553/accreditation-standards-for-hospitals-in-lebanon-january-2019

Box 3: Process adopted and impact achieved for the topic of addressing medical errors in the health system **Issue**: There is insufficient attention to quality and patient safety in healthcare; no explicit national policy for quality improvement and patient safety that sets out goals and indicators, clarifies roles and responsibilities, and identifies incentives and non-incentives across the entire healthcare spectrum.

**Priority setting**: Focusing event (wide media coverage of a 9-month old child who underwent double amputation as a result of a medical error). Subsequent discussions with policymakers from the Ministry of Public Health led to refinement of topic as follows: Effectiveness of interventions to address medical errors in the healthcare system.

**Evidence synthesis**: Identification and leveraging of preexisting systematic reviews addressing the prioritized topic.

**Knowledge translation product**: Policy brief titled “Addressing medical errors in the Lebanese healthcare system.” Findings from the identified systematic reviews as well as other relevant global and local research evidence were incorporated into the policy brief, and subsequently contextualized to the Lebanese health system. Key policymakers and stakeholders were involved in shaping and framing the outline for the policy brief.

**Knowledge uptake activities**: Policy dialogue; post-dialogue summary report; personalized debriefings; follow-up on implementation through the establishment of national standards for patient safety including training requirements for the hospital accreditation program.

**Impact:**

- Incorporation of patient safety goals, indicators, and training requirements in the national accreditation standards of the hospital accreditation programs in Lebanon
- Revision of the contractual agreements with healthcare organizations in Lebanon to incorporate selected quality indicators as part of the reimbursement formula

**Reference Document:** The Ministry of Public Health announced the official publication of the new accreditation standards manual for hospitals. https://www.moph.gov.lb/en/Pages/3/20553/accreditation-standards-for-hospitals-in-lebanon-january-2019

Box 4: Process adopted and impact achieved for the topic of integrating palliative care into the health system **Issue**: The high burden of non-communicable diseases (NCDs) and the rapidly aging population in Lebanon makes palliative care (PC) an essential component of health services needed to relieve the suffering of patients. In Lebanon, the demand for PC is expected to increase with the aging population and the high burden of NCDs. In 2015, Lebanon had the highest percentage of people aged 65 years and older (7.3%) in the Arab region and this number is projected to reach 12% by the year 2030.

http://nna-leb.gov.lb/en/show-news/93313/nna-leb.gov.lb/en

**Status quo**: Palliative care services in Lebanon are not accessible to a wide range of patients due to barriers at the organizational, system, professional, and patient levels. PC services are not financially covered by public and most private insurance parties. In addition, Lebanon does not have a national PC law or a stand-alone national plan to support PC development and integration.

**Priority setting**: Stakeholder-driven (push from NGOs).

**Evidence synthesis**: Identification and leveraging of preexisting systematic reviews addressing the prioritized topic.

**Knowledge translation product**: Policy brief titled “Integrating Palliative care into the Health System.” Findings from the existing systematic reviews in addition to other global and local research evidence were incorporated into the policy brief, and subsequently contextualized to the Lebanese health system. Key policymakers and stakeholders were involved in shaping and framing the outline for the policy brief.

**Knowledge uptake activities:** Policy dialogue; post-dialogue summary report; and advocacy through coalition of NGOs.

**Impact:**

Issuance of a Ministerial Decree on reimbursement for palliative care. On 18 March, the Ministry of Public Health took a major step toward integrating palliative care into the Lebanese health system by issuing Decree 1/447 that defines criteria for the reimbursement of palliative care services. The decree defines coverage for both home- and hospital-based programs and provides a blueprint for a reimbursement structure that can be applied by the National Social Security Fund (NSSF) and private insurers.

**Reference Document**: https://www.executive-magazine.com/economics-policy/lebanons-ministry-of-public-health-issues-decree-on-reimbursement-for-palliative-caresystem

### Factors influencing uptake of evidence

Our interpretive assessment of changes to public policy process, according to Kingdon’s streams theory, revealed that the convergence of the problem, policy, and politics streams opened a window of opportunity to bring the issue to the decision agenda, propelling government action (Table 3). The problem streams were largely shaped by focusing events or donors’ and stakeholders’ interests; the policy streams by availability of policy alternatives to address the problems; and the politics streams by national mood, pressure from interest groups, and alignment with governmental reforms.

**Table 3:** Application of Kingdon’s model to the case studies

Factors	Syrian refugees	Medical errors	Palliative care	Pharmaceuticals
Problem	Political event: In 2013, Lebanon was hosting 1.1 million Syrian refugees, the highest per capita refugee population in the world. These refugees faced a high burden of disease, and the obligation to address their health needs has put substantial pressure on the Lebanese health care system, particularly in terms of access, cost, and quality	Focusing event: In 2015, the issue of medical error gained tremendous attention in Lebanon following public outcry and extensive media coverage of the case of a 5-year-old child who underwent double amputation as a result of a medical error	Local report: According to a report published in 2017, an estimated number of 15,000 patients need palliative care services each year in Lebanon. This number is expected to increase due to a number of factors, such as the aging population and the rise in NCDs; yet, current health system arrangements do not ensure adequate access to palliative care services	This is a broad problem facing society, which became identified as issues that require public attention in a formal priority setting meetings. Primary studies indicate problems with the quality and prescribing of pharmaceutical drugs in Lebanon, which puts patients at risk of serious adverse effects, increases drug resistance, and leads to unnecessary increased costs on patients and the health system
Policy	Evidence demonstrated limited coordination between organizations and agencies providing health services to refugees, which was leading to both duplication and gaps in delivery of those services Generation of a set of policy alternatives to address the problem (through a briefing note document that was circulated to stakeholders)	Evidence demonstrated while there has been an increase in reporting of medical errors in Lebanon, the associated implications and debates about causes, responsibilities, and accountabilities are ill-informed, and in many cases, not leading to real improvement in patient safety practices Generation of a set of policy alternatives to address the problem (through a policy brief document that was circulated to stakeholders)	Evidence demonstrated failure of existing paradigm Generation of a set of policy alternatives to address the problem (through a policy brief document that was circulated to stakeholders)	Evidence demonstrated inadequate measures at the policy, organizational, healthcare professional, and consumer levels to promote quality of pharmaceuticals Generation of a set of policy alternatives to address the problem (through a policy brief document that was circulated to stakeholders)
Politics	Shift in national mood (including change in political climate, prevailing public opinion, and funder organizations’ agendas) favored immediate action on this issue	Shift in the balance of organized forces (i.e., interest group pressure campaigns through the public, advocates, and the media) coupled by demand and commitment from policymakers stimulated policy change	Policy champions (through well-established NGOs and stakeholder organizations) and ongoing health sector reform plan at that time, which presented an opportunity to shape the policy agenda around this issue	Policy champion (one of the policymakers was working on Codes of Ethics for Medicinal Product Promotion) and shift in the balance of organized forces (i.e., interest group pressure campaigns through media)

## Discussion

To our knowledge, this is the first study to describe both the development and implementation of an impact-oriented approach to link evidence synthesis to policy in the EMR. The approach incorporates three phases: (1) priority setting, (2) evidence synthesis, and (3) uptake. It encourages researchers, policymakers, and stakeholders to interact at each phase to achieve the desired impact. The approach can support evidence-informed policymaking and practice by aligning evidence synthesis production to policy priorities, fostering interactions between researchers and policymakers, and utilizing a range of knowledge translation and uptake activities to achieve policy impact and action [20, 41–43]. Additionally, by considering the appropriateness of producing evidence synthesis products, particularly systematic reviews, the approach responds to growing calls to make research more valuable by avoiding duplication of research and reducing research waste [44, 45]. The application of this approach is of particular relevance to low- and middle-income countries, including the EMR, where evidence syntheses are limited and often misaligned with policy needs and priorities, and with inadequate efforts to promote their uptake and use in policymaking and practice [15, 16, 46, 47].

Moreover, the approach recognizes that evidence-informed health policymaking is a complex phenomenon, and that contextual factors need to be taken into consideration if evidence is to be influential; in doing so, it contests the simple, linear conceptualization of the relationship between research and policy, built upon a singular input of research and independently of policy or politics [48, 49]. As illustrated by the approach, what is being supported is a synthesis of body of knowledge, undertaken by researchers embedded in the political context in which they operate, and packaged and contextualized to achieve impact. Far from being isolated from politics, the case studies and the use of Kingdon’s model acknowledge that policymaking occurs via macro-analysis involving several actors, organizations, institutions, and activities, all of which affect the end result. The approach also recognizes that policymaking processes should be strengthened through the building of organizational capacity and supporting institutional infrastructures to facilitate access, appraisal, and application of evidence synthesis to policy.

Application of the approach has generated some important lessons to optimize its use and promote evidence-informed policymaking and practice within and beyond Lebanon. Below, we reflect on some of the key lessons learned:

*Build trust and create demand for evidence* Lack of trust can be an initial barrier for policymakers to engage with evidence, thus highlighting the need to challenge existing preconceptions and build confidence in evidence-informed health policymaking and practice [50]. Building trust with policymakers can be achieved through demonstrating high degree of responsiveness to their priorities and needs, maintaining continuous and close relationships with them, managing demand and expectations, remaining objective and politically neutral, and maintaining credibility. Creating demand for evidence can occur through enhancing awareness of policymakers on the importance of evidence-informed health policymaking and practice; building capacities in accessing and using evidence; and engaging policymakers in different steps of the process. Importantly, anticipating the demand for evidence and influencing such demand presents an important opportunity to demonstrate proof of concept and thus create demand for evidence. This was the case for the medical error case study whereby strong media coverage and public outcry pressured the government to act, thus opening a window of opportunity to push the issue of quality and safety of healthcare to the forefront of policymakers’ agendas.

*Engage policymakers and stakeholders early on and ensure their engagement throughout the process (co-production approach)* Engaging policymakers and stakeholders at each step of the approach is critical to ensuring relevance, accessibility, and uptake of evidence in policymaking and practice. In all four case studies, deliberate decisions were made to engage key national policymakers, professional associations, nongovernmental organizations, and other stakeholders in each phase from shaping priorities, to framing the evidence synthesis topics, to refining the outline for knowledge translation products, to contextualizing policy options in the policy dialogue, and finally, impact assessment. This level of engagement helps create a sense of ownership and increases commitment of policymakers and stakeholders, which in turn increases the likelihood of the evidence being used in policymaking and practice. The COVID-19 pandemic further re-enforced the importance of co-production for policymaking, where researchers work together with knowledge users (made up of policy makers, health system leaders, clinicians, patients, the public, and others) to identify a problem and produce knowledge, sharing power and responsibility from the start to the end of the research [51] Although patient advocacy groups were presented at some of the dialogues presented in the case studies, it may be worthy, in future initiatives, to consult citizens and patients through focus groups designed specifically for citizens and patients, allowing them to voice their true opinions and concerns. This is an area that K2P Center is currently working on.

*Align research production with policy priorities (and identify window of opportunity for action)* To generate research that can inform and guide policy, it is critical to link the production of evidence syntheses to the needs and priorities of policymakers. Priority setting is the first, and often missing, step in connecting evidence syntheses with policy needs. Furthermore, recognizing a window of opportunity for action (e.g., receptiveness of political climate for change, alignment with key international events, and reform initiatives) is another critical factor to increasing policy relevance, and thus the subsequent uptake of evidence in policymaking.

*Evidence synthesis alone is unlikely to lead to impact; contextualization and dissemination of actionable evidence are critical* While systematic reviews or other syntheses of global evidence are increasingly recognized as a fundamental component of the evidence-informed approach to decision-making, there is growing realization that parameters of a single systematic review with one question may not always suffice to inform a national policy or program. Moreover, for systematic reviews and other evidence synthesis products to be policy-relevant and inform decisions, they need to be packaged in user-friendly format, communicated in an easy-to-understand language, and contextualized to local needs and constraints. This highlights the importance of knowledge translation products, which combine insights from multiple sources to provide context-specific and actionable evidence to enable countries to adapt global solutions to local needs and constraints.

*Deliberative stakeholder dialogues are highly valued knowledge uptake activities, but they may not always be sufficient to achieve impact* Our experience reinforces the premise of a deliberative dialogue (informed by knowledge translation products) as a strategy for supporting evidence-informed policies and practice [52]. Respondents consistently perceived the policy briefs and dialogues very favorably and indicated strong intention to use research evidence, regardless of the issue or group involved. Nonetheless, to sustain momentum created during the dialogue, it may be necessary to engage in additional post-dialogue activities. The dissemination of post-dialogue summary documents that provide a roadmap for action is one way to do so. Personalized debriefings with key targeted policymakers and stakeholders, particularly those who have a strong say about the issue, is another important strategy to reinforce commitment and follow through with the implementation of the agreed upon action plan emerging from the policy dialogue. Our experience also suggests that when applied properly, media engagement can have a positive role in mobilizing public opinion, raising awareness, and pushing for policy change. This aligns with findings from a systematic review on the role of media interventions in influencing health policymaking process [53]. Evidence-based advocacy can also be considered for contentious and politically charged topics, as was the case with the topic on promoting pharmaceutical quality, where there was significant resistance from pharmaceutical companies and physicians interacting with them. In such an instance, advocacy can help increase stakeholder support for the policy change, create a public will to push the issue on the policy agenda, and create a coalition to develop, implement, and evaluate advocacy tactics to support implementation of a given policy or action.

*Extend support to policy implementation* Successful policy outcomes depend not only upon designing good policies, but also upon managing their implementation [54]. Traditionally, implementation has been assumed to be a routine function; once decision-making is made, it will be carried out smoothly. This top-down approach to implementation is proving to be challenging, given the multitude of social, economic, technological, and political contexts that affect implementation. Rather than just let policies drift into full or even partial failure, there is a growing realization that support should be extended to policy implementation by involving policy and program implementers in the design and execution of the policy or program while taking into consideration existing organizational goals, strategies, and incentives. Additionally, identifying barriers to implementation at different levels, such as recipients of care, providers of care, health system constraints, and social and political constraints, as well as providing counterstrategies to overcome those barriers, can increase the likelihood of effective implementation. In the case of Syrian refugees and medical error, the generation of materials to support the implementation process facilitated the achievement of impact.

*Impact is the driving force: make sure to measure it* Integrating monitoring and evaluation early on enables teams to measure whether and how evidence was used in policy and practice, and whether the policy achieved its intended outcome and impact [55]. While the 6-month period between the dialogue and the post-dialogue survey is appropriate for examining short-term developments, a longer period might be needed to capture further changes that might have materialized at later stages. Prospective policy tracing that combines documentary review, media analysis, and interviews with key selected stakeholders can be carried out to assess whether important policy and practice actions have been undertaken concerning the policy issue [56].

*Establish linkages to complementary organizations within the evidence ecosystem* No one entity may possess all skills and competencies needed for successful implementation of the approach to achieve impact. Building bridges to other complementary organizations within the evidence ecosystem produces teams of people with different backgrounds, perspectives, and complementary skills. The unique collaboration between SPARK and the K2P Center allowed for the adoption of a holistic approach spanning from priority setting to evidence synthesis, uptake, and impact assessment. Leveraging of common resources, capacities, and expertise enhanced the feasibility and effectiveness of the approach and facilitated evidence-informed health policymaking and practice.

### Strengths and limitations

A strength of this study is that a multidisciplinary team was involved in the development of the approach with input of international experts in evidence synthesis, knowledge translation, and evidence-informed policymaking. As the first of its kind in the EMR, the study demonstrated the utility and applicability of the approach to this context. We used multiple case studies, drawing on different data sources to examine the influence of the approach on policy and capture impact. Having said this, it is important to caution that this study does not claim a cause–effect relationship emerging from the presented approach. As indicated, other factors may have also contributed to the positive changes that followed, such as recognition and interest of policymakers in the issue, alignment with existing health system reforms, and efforts made by different researchers and civil society groups to advance the issues in the country. The application of Kingdon’s model helped explain how key contextual factors facilitated the ascendancy of the issues to the health agenda.

There are some limitations worth acknowledging, many of which reflect existing gaps in the science of knowledge translation as opposed to the design and execution of the study.

While there is still controversy about what endpoints should be considered when it comes to impact and how they should be measured, the explicit use of evidence in the policymaking process (recognizing the range of other influential factors to be considered in the process) is a commonly used outcome [57, 58]. As such, we applied multiple case studies to examine the influence of our approach in policy, while drawing on interviews, documentary analyses, and previously collected data (from surveys) to produce the case descriptions and document the impact. Triangulation of data sources allowed for cross-checking and validation of findings.

Moreover, while a single case study cannot represent all of the distinctions in approach available, our choice of diverse policy cases improved the generalizability of these results to other policy cases in Lebanon and the region. Convergence of findings from the different case studies further served as a form of validation. Nonetheless, the approach could benefit from further testing and application in other contexts to assess its generalizability as well as generate more empirical data to weigh the various components of the approach, or attribute the presence or absence of any of those components to the degree of impact. Our study and similar types of publications will serve to strengthen the science of knowledge translation, where persistent gaps continue to exist.

Finally, for two of the case studies, we could not evaluate the policy briefs and the deliberative dialogues; however, we believe the findings are representative of policymakers’ perceptions in Lebanon, especially since they also align with findings from the literature across settings and topics [52, 59, 60].

### Implications for research and practice

Although the path of promoting evidence-informed policies and practice is a long and winding road in the EMR [21, 61, 62], the COVID-19 pandemic has opened substantial windows of opportunity for accelerating action toward evidence-informed policymaking with emphasis on providing access to well-packaged, relevant, and updated synthesis of the best available evidence on priority topics in a timely manner [6, 63]. Study findings can inform the work of researchers, governments, and funders seeking to promote evidence-informed health policymaking and practice in their own context.

Future research can seek to test and adapt the approach to different contexts (e.g., low-income countries) and situations (e.g., non-health sector issues or pandemic response) to support evidence-informed policymaking and practice. In particular, the COVID-19 pandemic has highlighted the importance of timeliness of evidence, thus it may be worthy, in future initiatives, to experiment with rapid response products and ways to expedite the impact-oriented approach to provide timely responses to emerging policy priorities. In fact, both SPARK and K2P Center are scaling up their activities to provide rapid response services. More research is also needed to explore which combination of strategies and tools are most effective and in what contexts, and the relative attribution of the different components of the approach to impact.

There is also a need for more research on rigorous methods and frameworks for evaluating the impact of research (and evidence synthesis) on both policy and populations, with conceptual clarity and metrics for the variety of impacts leading from research (e.g., better access to care and improved health outcomes). Strengthening the research base on impacts would solidify the argument for promoting evidence-informed policies and practice.

More research is also warranted on how to embed and institutionalize the use of knowledge approaches (like the one developed in this study) within policymaking processes to ensure sustainability of evidence-informed policies and practice.

## Conclusions

The impact-oriented approach linking evidence synthesis to policy will address a gap in the scientific literature. We believe the approach will be useful for researchers, governments, and funders seeking to promote policy-relevant evidence syntheses. It will help support evidence-informed policymaking and practice and reduce research waste by aligning evidence synthesis production to policy priorities, fostering researcher–policymaker interactions, and harnessing knowledge translation products and uptake activities to package, contextualize, and promote the use of evidence to achieve impact. We encourage people involved in evidence-informed policymaking to use the approach and systematic reviewers and knowledge translation platforms to conduct further testing within their own contexts as a contribution to further refining the approach.

## Data Availability

All data generated or analyzed during this study are included in this published article [and its supplementary information files].

## Footnotes

1 http://2014.colloquium.cochrane.org/workshops/prioritizing-questions-systematic-reviews-health-policy-and-systems-research.

2 http://www.who.int/alliance-hpsr/news/symposiumschedfinal3.pdf.

## References

[1] Koon AD, Rao KD, Tran NT, Ghaffar A (2013). Embedding health policy and systems research into decision-making processes in low- and middle-income countries. Health Res Policy Syst.

[2] World Health Organization. Evidence-informed decision-making for health policy and programmes: insights and best practices from successful country initiatives. 2021.

[3] Pantoja T, Barreto J, Panisset U (2018). Improving public health and health systems through evidence informed policy in the Americas. BMJ.

[4] Chhetri DZF (2021). Advocacy for evidence-based policy-making in public health: experiences and the way forward. J Health Manag.

[5] Rodriguez DC, Shearer J, Mariano AR, Juma PA, Dalglish SL, Bennett S (2015). Evidence-informed policymaking in practice: country-level examples of use of evidence for iCCM policy. Health Policy Plan.

[6] Stewart R, El-Harakeh A, Cherian SA (2020). Evidence synthesis communities in low-income and middle-income countries and the COVID-19 response. Lancet.

[7] Gough D, Davies P, Jamtvedt G, Langlois E, Littell J, Lotfi T (2020). Evidence Synthesis International (ESI): position statement. Syst Rev.

[8] London School of Hygiene & Tropical Medicine. Evidence Synthesis https://www.lshtm.ac.uk/research/centres/centre-evaluation/evidence-synthesis.

[9] Gough D, Oliver S, Thomas J (2017). An introduction to systematic reviews.

[10] Moher D, Stewart L, Shekelle P (2015). All in the family: systematic reviews, rapid reviews, scoping reviews, realist reviews, and more. Syst Rev.

[11] Pearson A (2014). Evidence synthesis and its role in evidence-based health care. Nurs Clin.

[12] Donnelly CA, Boyd I, Campbell P, Craig C, Vallance P, Walport M (2018). Four principles for synthesizing evidence. Nature.

[13] Oliver S, Bangpan M, Dickson K (2017). Producing policy relevant systematic reviews: navigating the policy-research interface. Evidence and Policy..

[14] Langlois ÉV, Akl. EA. Fostering the use of evidence synthesis findings in policy and practice. World Health Organization; 2018. 33877749

[15] El-Jardali F, Akl EA, Karroum LB, Kdouh O, Akik C, Fadlallah R (2014). Systematic reviews addressing identified health policy priorities in Eastern Mediterranean countries: a situational analysis. Health Res Policy Syst.

[16] Akkawi A, Khabsa J, Noubani A, Jamali S, Sibai AM, Lotfi T (2020). Non-communicable diseases research output in the Eastern Mediterranean region: an overview of systematic reviews. BMC Med Res Methodol.

[17] Law T, Lavis J, Hamandi A, Cheung A, El-Jardali F (2012). Climate for evidence-informed health systems: a profile of systematic review production in 41 low- and middle-income countries, 1996–2008. J Health Serv Res Policy.

[18] Chaabna K, Cheema S, Abraham A, Maisonneuve P, Lowenfels AB, Mamtani R (2021). The state of population health research performance in the Middle East and North Africa: a meta-research study. Syst Rev.

[19] El-Jardali F, Lavis JN, Ataya N, Jamal D, Ammar W, Raouf S (2012). Use of health systems evidence by policymakers in eastern Mediterranean countries: views, practices, and contextual influences. BMC Health Serv Res.

[20] Hyder AA, Corluka A, Winch PJ, El-Shinnawy A, Ghassany H, Malekafzali H (2011). National policy-makers speak out: are researchers giving them what they need?. Health Policy Plan.

[21] El-Jardali F, Mandil A, Jamal D, BouKarroum L, El-Feky S, Nour M (2018). Engagement of health research institutions in knowledge translation in the Eastern Mediterranean Region. East Mediterr Health J.

[22] El-Jardali F, Lavis JN, Ataya N, Jamal D (2012). Use of health systems and policy research evidence in the health policymaking in eastern Mediterranean countries: views and practices of researchers. Implement Sci.

[23] Ward V, House A, Hamer S (2009). Developing a framework for transferring knowledge into action: a thematic analysis of the literature. J Health Serv Res Policy.

[24] Milat AJ, Li B (2017). Narrative review of frameworks for translating research evidence into policy and practice. Public Health Res Pract.

[25] Wensing M, Grol R (2019). Knowledge translation in health: how implementation science could contribute more. BMC Med.

[26] McKee M (2019). Bridging the gap between research and policy and practice comment on “CIHR Health System Impact Fellows: Reflections on ‘Driving Change’ Within the Health System”. J Health Policy Manag.

[27] Huybrechts I, Declercq A, Verté E, Raeymaeckers P, Anthierens S (2021). The building blocks of implementation frameworks and models in primary care: a narrative review. Front Public Health.

[28] Nilsen P (2015). Making sense of implementation theories, models and frameworks. Implement Sci.

[29] El-Jardali F, Fadlallah R (2015). A call for a backward design to knowledge translation. Int J Health Policy Manag.

[30] Kim C, Wilcher R, Petruney T, Krueger K, Wynne L, Zan T (2018). A research utilisation framework for informing global health and development policies and programmes. Health Res Policy Syst.

[31] World Bank. The World Bank In Lebanon. 2022.

[32] Lavis JN, Permanand G, Oxman AD, Lewin S, Fretheim A (2009). SUPPORT Tools for evidence-informed health Policymaking (STP) 13: Preparing and using policy briefs to support evidence-informed policymaking. Health Res Policy Syst.

[33] Silva MJD, Breuer E, Lee L, Asher L, Chowdhary N, Lund C (2014). Theory of Change: a theory-driven approach to enhance the Medical Research Council's framework for complex interventions. Trials.

[34] Walt G, Shiffman J, Schneider H, Murray SF, Brugha R, Gilson L (2008). “Doing” health policy analysis: methodological and conceptual reflections and challenges. Health Policy Plan.

[35] Kingdon JWSE (1984). Agendas, alternatives, and public policies.

[36] Akl EA, Fadlallah R, Ghandour L, Kdouh O, Langlois E, Lavis JN (2017). The SPARK Tool to prioritise questions for systematic reviews in health policy and systems research: development and initial validation. Health Res Policy Syst.

[37] Fadlallah R, El-Harakeh A, Bou-Karroum L, Lotfi T, El-Jardali F, Hishi L (2020). A common framework of steps and criteria for prioritizing topics for evidence syntheses: a systematic review. J Clin Epidemiol.

[38] In: Langlois EV, Daniels K, Akl EA, editors. Evidence synthesis for health policy and systems: a methods guide. Geneva. 2018. 33877749

[39] Lavis JN, Boyko JA, Oxman AD, Lewin S, Fretheim A (2009). SUPPORT Tools for evidence-informed health Policymaking (STP) 14: organising and using policy dialogues to support evidence-informed policymaking. Health Res Policy Syst..

[40] Oliver K, Lorenc T, Innvaer S (2014). New directions in evidence-based policy research: a critical analysis of the literature. Health Res Policy Syst.

[41] Chapman E, Pantoja T, Kuchenmuller T, Sharma T, Terry RF (2021). Assessing the impact of knowledge communication and dissemination strategies targeted at health policy-makers and managers: an overview of systematic reviews. Health Res Policy Syst.

[42] Lavis JN, Lomas J, Hamid M, Sewankambo NK (2006). Assessing country-level efforts to link research to action. Bull World Health Organ.

[43] Maleki K, Hamadeh RR, Gholami J, Mandil A, Hamid S, Butt ZA (2014). The knowledge translation status in selected Eastern-Mediterranean universities and research institutes. PLoS ONE.

[44] Chalmers I, Bracken MB, Djulbegovic B, Garattini S, Grant J, Gulmezoglu AM (2014). How to increase value and reduce waste when research priorities are set. Lancet.

[45] Ioannidis JP, Greenland S, Hlatky MA, Khoury MJ, Macleod MR, Moher D (2014). Increasing value and reducing waste in research design, conduct, and analysis. Lancet.

[46] Eliea A, Tamara L, Fadi E-J, Langlois ÉV. Addressing challenges in the conduct of policy-relevant evidence syntheses 2018

[47] Uneke CJ, Sombie I, Johnson E, Uneke BI, Okolo S (2022). Promoting the use of evidence in health policy-making in the economic commission of the West African States Region: exploring the perception of policy-makers on the necessity of an evidence-based policy-making guidance. Ann Afr Med.

[48] Boswell C. Smith K. Rethinking policy ‘impact’: four models of research-policy relations. Palgrave Communications. 2017;3(44).

[49] Bowen S, Zwi AB (2005). Pathways to “evidence-informed” policy and practice: a framework for action. PLoS Med.

[50] Bowen S, Martens P (2005). Demystifying knowledge translation: learning from the community. J Health Serv Res Policy.

[51] Marten R, El-Jardali F, Hafeez A, Hanefeld J, Leung GM, Ghaffar A (2021). Co-producing the covid-19 response in Germany, Hong Kong, Lebanon, and Pakistan. BMJ.

[52] Partridge ACR, Mansilla C, Randhawa H, Lavis JN, El-Jardali F, Sewankambo NK (2020). Lessons learned from descriptions and evaluations of knowledge translation platforms supporting evidence-informed policy-making in low- and middle-income countries: a systematic review. Health Res Policy Systems.

[53] Bou-Karroum L, El-Jardali F, Hemadi N, Faraj Y, Ojha U, Shahrour M (2017). Using media to impact health policy-making: an integrative systematic review. Implement Sci.

[54] Ar KHAN (2016). Policy implementation: some aspects and issues. J Community Posit Pract.

[55] Kuchenmuller T, Chapman E, Takahashi R, Lester L, Reinap M, Ellen M (2022). A comprehensive monitoring and evaluation framework for evidence to policy networks. Eval Program Plann.

[56] World Health Organization. Evidence briefs for policy: using the integrated knowledge translation approach: guiding manual. World Health Organization. Regional Office for Europe; 2020.

[57] Dobbins M, Hanna SE, Ciliska D, Manske S, Cameron R, Mercer SL (2009). A randomized controlled trial evaluating the impact of knowledge translation and exchange strategies. Implement Sci.

[58] Welch VA, Petticrew M, O'Neill J, Waters E, Armstrong R, Bhutta ZA (2013). Health equity: evidence synthesis and knowledge translation methods. Syst Rev.

[59] Ogbonnaya LU, Okedo-Alex IN, Akamike IC, Azuogu B, Urochukwu H, Ogbu O (2021). Assessing the usefulness of policy brief and policy dialogue as knowledge translation tools towards contextualizing the accountability framework for routine immunization at a subnational level in Nigeria. Health Res Policy Syst.

[60] Moat KA, Lavis JN, Clancy SJ, El-Jardalid F, Pantojae T (2013). Evidence briefs and deliberative dialogues: perceptions and intentions to act on what was learnt. Bull World Health Organ.

[61] Rabbata ME, El-Jardali F, Fadlallah R, Sorord S, Ahmadnezhade E, Badrf E (2021). Funding for health policy and systems research in the Eastern Mediterranean region: amount, source and key determinants. Public Health Res Pract..

[62] Haq Z, Hafeez A, Zafar S, Ghaffar A (2017). Dynamics of evidence-informed health policy making in Pakistan. Health Policy Plan.

[63] WHO. Evidence as a catalyst for policy and societal change. Towards more equitable, resilient, and sustainable global health. 2021. https://www.e2psummit2021.org/.

